# Pain management in patients with end-stage renal disease and calciphylaxis- a survey of clinical practices among physicians

**DOI:** 10.1186/s12882-020-02067-2

**Published:** 2020-09-18

**Authors:** Rajkumar Chinnadurai, Smeeta Sinha, Aoife C Lowney, Mary Miller

**Affiliations:** 1 Department of Renal Medicine, Salford Royal NHS Foundation Trust, Salford, M6 8HD UK; 2 Palliative Medicine, Sir Michael Sobell House Hospice, Oxford, UK; 3 Palliative Medicine, Oxford University Hospitals NHS Foundation Trust, Oxford, UK

**Keywords:** Calciphylaxis, Guidelines, Opioids, Pain management, Palliative care, Survey

## Abstract

**Background:**

Calciphylaxis is a rare condition usually seen in patients with end-stage renal disease. Pain is a hallmark of this condition and can be extremely difficult to control. Anecdotal data suggests that pain management in calciphylaxis is challenging with variable approaches across the United Kingdom (UK) and internationally. A knowledge and practice survey was conducted to establish current practice in the management of pain in patients with calciphylaxis, in the UK. Based on the results and clinical experience the authors suggest a clinical practice guideline.

**Methods:**

An online questionnaire was circulated among physicians (renal and palliative care) involved in the management of pain in calciphylaxis. The questionnaire included a mix of open-ended questions and questions with drop down options.

**Results:**

One hundred and six clinicians responded to the survey of which 60 (57%) respondents were from palliative medicine; the remaining 46 (43%) were from renal medicine. 31 (30%) respondents across both specialties had not encountered any patients with a diagnosis of calciphylaxis (renal-2, palliative care-29). A referral to the palliative care team was undertaken by 18% of renal physicians, 32% referred to the pain team and 50% referred to both. Only 3% of the palliative medicine respondents indicated that they had received a referral from the renal team at the time of diagnosis. Opioids were the preferred initial drug of choice for the management of all types of pain. Paracetamol was universally selected as the preferred first-choice adjuvant agent for management of all types of pain. The importance of advance care planning was highlighted with 72% undertaking advanced care planning discussions often or most of the time.

**Conclusion:**

There was wide variation in the current practice of pain management in patients with calciphylaxis, with variation between renal specialists and palliative care specialists. Referral to specialists in pain management is not universal despite the severe nature of the pain experienced by patients with calciphylaxis. The data generated has facilitated the development of a clinical practice guideline to support complex pain management in a group of patients with multiple comorbidities.

## Background

Calcaemic uremic arteriolopathy or calciphylaxis is a rare but life-threatening condition usually seen in patients with end-stage renal disease (ESRD). The reported incidence in haemodialysis patients is variable and largely reliant on registry data with reported incidences of 0.04% in Germany and 0.35% in the United States. The mortality rate can be as high as 60–80% [1]. It is characterised by the onset of a painful violaceous rash which progresses to ischaemic necrotic ulcers. The lesions are predominantly centrally distributed in areas of high adiposity (e.g. abdomen and thighs) [2].

Pain is a hallmark of this disease and can be extremely difficult to control. Pain resulting from calciphylaxis is classically an acute ischaemic pain resulting from tissue damage as a consequence of arteriolar occlusion causing hypoperfusion, ischaemia and infarction of tissues [3]. As with all acute pain the severity and characteristics of the pain will vary from patient to patient. The acute pain may be a continuous background pain or evoked by movement or procedures. In addition to the background level of pain there may be episodes of unprovoked exacerbations of breakthrough pain. Opioids have been a key component in managing pain.

Current management of calciphylaxis involves a multimodal approach including medical (stopping medications which have been associated with increased risk e.g. calcium-based phosphate binders, vitamin D analogues, vitamin K antagonists, and starting new medication e.g. sodium thiosulphate, cinacalcet, and antibiotics), surgical (e.g. parathyroidectomy, wound debridement, and amputation) and modification of dialysis regimens such as varying dialysate calcium concentrations and increasing dialysis frequency [4–8].

Available evidence suggests that pain management in calciphylaxis is unsatisfactory with differing practice across the United Kingdom (UK) and internationally [9]. A knowledge and practice survey was conducted to obtain information on physicians current practice of the management of pain in patients with calciphylaxis in the UK and the Republic of Ireland. The aim of the data collection was to describe practice patterns and in light of this data develop a practice guideline for pain management in patients with calciphylaxis. The guidelines described are the first step in developing a rationale for managing complex pain in a group of frail patients with multiple comorbidities.

## Methods

An online questionnaire was circulated among renal physicians and palliative medicine physicians in the UK and Ireland to conduct a qualitative survey. (https://www.gmann.co.uk/website/calciphylaxis-pain-management-survey.cfm). The survey was distributed to the palliative care physicians through the Association of Palliative Medicine, the only membership organisation in the UK and Republic of Ireland. The National Registry of Rare Kidney Diseases (RaDaR) distributed the survey to the renal physicians. A reminder was sent out once via both organisations. Questions included a mix of open-ended questions and questions with drop down options (Additional file 1). The questionnaire was formulated to elucidate perceptions and practice in the management all three types of pain in calciphylaxis: background, breakthrough and procedure related pain. In addition, questions were framed to capture practice regarding the type and the route of analgesia used. All free text comments were reviewed by the four authors but given the lack of uniformity they did not contribute any additional value to the survey, so they were not added to the survey (Additional file 2). The data was transferred to a Microsoft excel workbook and analysed. This project was assessed by the Research & Innovation team at the Northern Care Alliance NHS Group, against the relevant guidelines, and did not require an ethical review.

## Results

**Fig. 1:**
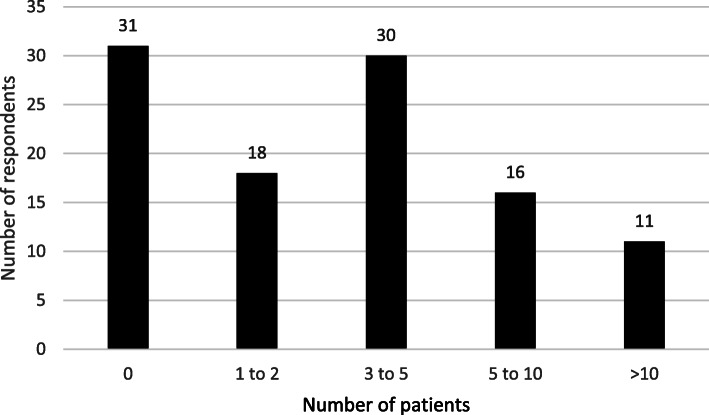
Number of patients with a diagnosis of calciphylaxis managed in past 10 years

One hundred and six physicians responded to the survey, which is around 10% of the total distributed members. Of the total respondents, 60 (57%) were palliative medicine physicians and the remainder 46 (43%) were renal physicians. The respondents were predominantly (77%) working in acute care settings. Thirty percent of the respondents had not encountered any patients with calciphylaxis in the preceding 10 years, whilst 11 (10%) had managed more than 10 patients (Fig. 1).

The 31 (30%) who had no experience of managing patients with calciphylaxis were excluded from the subsequent analysis. Results presented are from 75 respondents (palliative medicine *n* = 31 and renal medicine *n* = 44). For the management of pain in patients with calciphylaxis, 8 (18%) of renal physicians refer to the palliative care team, 14 (32%) refer to the pain team and 22 (50%) refer to both specialties. Palliative care physicians almost universally (97%) described late referral patterns whereby referrals were made when the renal team were struggling to manage pain or when a patient was approaching end of life; only 3% received a referral at the time of diagnosis.

**Fig. 2:**
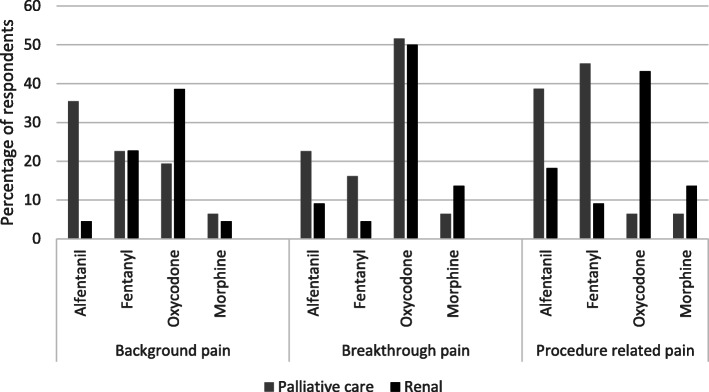
Opioid preference based on speciality

Opioids were the preferred drug of choice to manage pain. Oxycodone was the preferred (first choice) opioid for all types of pain (background, breakthrough and procedure related) with alfentanil and fentanyl being other popular choices (Table 1). Oxycodone was preferred by renal physicians whereas alfentanil and fentanyl were popular with palliative care physicians (Fig. 2). Most of the respondents (94%) report having diagnosed neurocognitive adverse effects when opioid analgesics were used.

**Table 1:** Preferred first choice opioid drug (number of respondents)

Drug	Background pain	Breakthrough pain	Procedure related pain
Oxycodone	24	38	21
Fentanyl	17	7	18
Alfentanil	13	11	23
Morphine	4	8	8
Tramadol	5	7	3
Codeine	6	2	0
Diamorphine	5	1	2
Buprenorphine	0	1	0
Methadone	0	0	0
Tapentadol	1	0	0

Both renal and palliative care physicians reported that modified release oral medications (73%) and continuous subcutaneous infusions of medications (51%) using a syringe driver were their preferred approaches to managing background pain. There was no clear drug preference for the management of breakthrough and procedure related pain with oral analgesia, subcutaneous analgesia or immediate release fentanyl preparations (lozenge, sublingual or nasal) being equally popular.

**Fig. 3:**
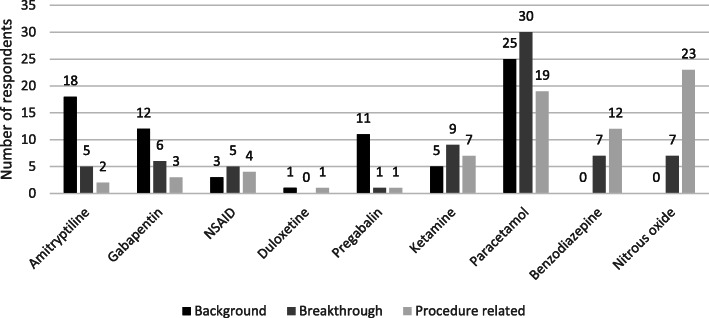
Preferred (first choice) adjuvant agents for pain management

Paracetamol was the preferred first-choice adjuvant agent for management of all types of pain, more so by renal physicians (Fig. 3 and Table 2). Five out of 75 respondents (6.6%) undertook other procedures such as epidural analgesia and nerve blocks to manage pain. Most of the respondents (83%) felt the presence of infection impacted on the effectiveness of analgesia.

**Table 2:** Preferred first-choice adjuvant agent for management of pain split by speciality (number of respondents)

Pain type	Drug	Total	Renal medicine	Palliative care
Background pain	Paracetamol	25	19	6
	Amitriptyline	18	11	7
	Gabapentin	12	9	3
	Pregabalin	11	4	7
	Ketamine	6	0	6
Breakthrough pain				
	Paracetamol	30	21	9
	Amitriptyline	5	2	3
	Gabapentin	6	4	2
	Ketamine	9	2	7
Procedure related pain				
	Paracetamol	19	17	2
	Benzodiazepine	12	6	6
	Nitrous oxide	23	11	12
	Ketamine	7	0	7

**Fig. 4:**
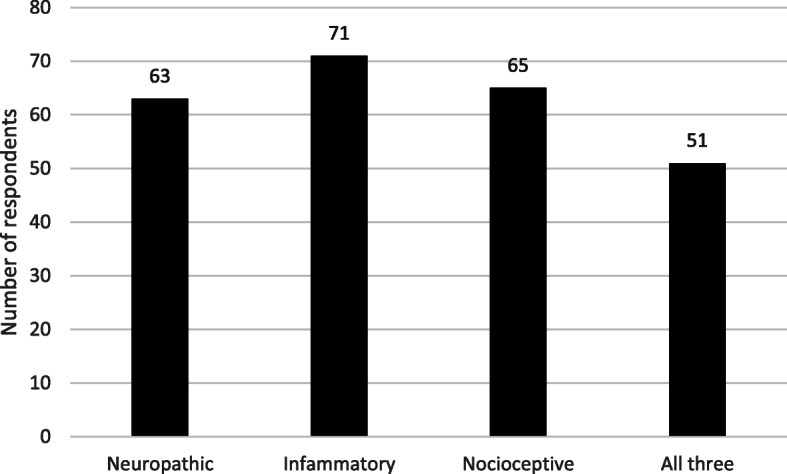
Types of pain in calciphylaxis

The perceived types of pain experienced by patients with calciphylaxis are illustrated in Fig. 4. The majority of respondents (72%), irrespective of speciality undertook advance care planning discussions often or most of the time. 28% of respondents used a pain scale, the most commonly used tool was the pain analogue scale (0–10) used by nine of the 20 respondents who used a tool (seven renal and two palliative care physicians).

## Discussion

Calciphylaxis is a rare and life-threatening condition associated with a significant impact on quality of life; pain is a major contributor to this. Due to the rarity and high mortality associated with this condition, randomised control trial evidence on management has been a challenge and there is no uniform strategy for the management of calciphylaxis associated pain.

Our survey demonstrated a wide variation in the management of pain in patients with Calciphylaxis. Notably the choice of medication varied depending on the speciality of the physician, indicating that familiarity with drugs and confidence in their use may be a factor in determining drug choice rather than an approach based on determining the nature of the pain and selecting the best choice of drug. Reported evidence in the literature on the management of pain in calciphylaxis is limited and variable. In a case series of three patients with Calciphylaxis, a cocktail approach of opioids, benzodiazepines and ketamine was used to manage refractory pain [3]. Similarly, a multistep approach including antidepressants, opioids and benzodiazepines was used with the involvement of palliative care in the management of a case of penile calciphylaxis [10]. Neurolytic lumbar sympathetic blockade has been used for the treatment of pain associated with calciphylaxis in ESRD patients [11]. In addition to analgesics, treatments that may improve the underlying lesion itself are also likely to improve pain. To date, there is no approved treatment for calciphylaxis, and management is largely based on case reports and uncontrolled studies, although prospective clinical trials are under way [12]. The use of hyperbaric oxygen therapy, vitamin K and wound debridement are examples of management strategies that been reported to improve wound healing and therefore may also improve pain.

Sodium thiosulphate (STS) has been widely reported as a potential treatment for calciphylaxis. The majority of the literature focuses on wound healing, however, Noureddine et al. reported that sodium thiosulphate attenuated pain in 71% of patients in a retrospective case series of 14 patients [13]. In a recent phase 2 trial SNF472, an intravenous formulation of myoinositol hexaphosphate has shown to improve wound healing, pain and quality of life in patients with calciphylaxis on haemodialysis [12]. Both SNF472 and STS are not traditional analgesics, however, the effect of these potentially disease modifying drugs on wound healing and consequently wound associated pain is an important patient reported outcome, which is now being reflected in the prominence of quantitative pain evaluation in clinical trial settings. A Phase 3 randomised double-blind placebo-controlled trial investigating the effect of STS on calciphylaxis associated pain is currently in progress (clinicaltrials.gov NCT03150420). The change in Pain Visual Analog Scale is a primary outcome measure in a Phase 3 randomised double-blind placebo-controlled trial investigating the effect of SNF472 on pain as well as wound healing (clinicaltrials.gov NCT04195906). The use of these agents within pain guidelines has not been routinely recommended as trial data is awaited.

Our survey also showed a variable approach to specialist referral to the pain or palliative care team. These findings are similar to observations in the literature. A systematic review of 12 calciphylaxis studies concluded that there was no formal study of palliative care in patients with calciphylaxis, illustrating the need for the development of a calciphylaxis specific patient-reported outcome measure which would help to prompt earlier and more frequent palliative care consultations [9]. The utility of palliative care consultations in the end of life care of 24 ESRD patients with calciphylaxis was reported by Olarinan et al. This study showed palliative care physicians were consulted in only 50% of the patients, with 54% receiving intensive care admission during the terminal phase of their illness. A very high rate of admission to intensive care despite the very poor prognosis highlights the need for a change in practice which promotes early referral, advance care planning and patient centred interventions [14]. Less than 10% of the members replied, which might be expected with a rare condition and may influence the results of the survey.

## Conclusion

In conclusion, there is wide variation in the current management of pain in calciphylaxis, amongst renal and palliative care physicians. The rarity of the condition and severity of pain are particularly challenging for non-pain specialists who have a tendency to revert to familiar drugs such as opioids. Moreover, there are no published guidelines or recommendations to support clinicians with decision-making.

## Recommendations

### Clinical practice recommendations for the management of calciphylaxis associated pain

Pain caused by calciphylaxis is nociceptive, neuropathic and inflammatory in nature as it is a result of metastatic microcalcification and tissue necrosis [15]. Choice of analgesia in patients with calciphylaxis is further complicated by ESRD and renal replacement therapy. The authors are not aware of any published guidance on pain management in this group. Therefore, the following guidance extrapolates from first principles and existing literature pertaining to the management of pain in patients with severe and end stage renal impairment but taking into account the unique challenge that calciphylaxis presents in terms of the severity of the pain as described by the physicians surveyed in this paper and the potential for opioid toxicity described by survey respondents as well as the expert opinion of the authors who are palliative medicine physicians (one working in renal palliative care) and renal physicians (one with an expertise in calciphylaxis) [16, 17].

The authors suggest the following pragmatic approach in this population.

1. Always refer to either the local palliative medicine team and / or pain team when calciphylaxis is suspected. This is because it is often a diagnosis of exclusion and pain control is difficult in this group and needs a specialist approach from the outset because of the additional morbidity associated with the use of inappropriate analgesia.
2. Tissue viability service/wound care specialist referral is essential and daily serial medical photography with consent is advised.
3. Ideally admit the patient to a renal unit or to a hospice for symptom control.
4. Where the patient has capacity, start advance care planning conversations. If the patient does not have capacity and that capacity cannot be enabled, start planning care using the principles of best interests. This is because of the high mortality associated with this condition. It is important to discuss the considerable risk of dying and poor prognosis with the patient and their family.
5. Consider enrolment in a clinical trial.

### Background pain control

If the patient is opioid naïve, commence a safe opioid at the lowest effective dose (select an opioid that does not have clinically relevant active metabolites that depend on renal excretion) and titrate to effect. The authors recognise that opioids are not considered appropriate for chronic pain. However, this is an acute pain arising in response to tissue injury and death [18] and therefore, we recommend an opioid as first line therapy.

In opioid naïve patients who are starting an opioid for the first time a starting dose of alfentanil 0.5 mg continuous subcutaneous infusion (CSCI) over 24 h is proposed. This is approximately equivalent to morphine 7.5 mg/24 h by subcutaneous infusion. The authors recommend alfentanil as it is not removed by dialysis, it does not cause toxicity in an ESRD population and it occupies a low volume in syringe drivers. This guidance is drawn from literature on the use of opioids in cancer patients with severe and end stage terminal illness [19–21] and is supported by the clinical pharmacology literature [18]. Fentanyl by CSCI is an alternative.

Where the patient is not opioid naïve, convert their existing opioid to alfentanil by CSCI. Palliative medicine team can help with conversions if this is outside of the usual scope of practice of the general clinician. Titrate daily to either pain relief or toxicity. Increases in background analgesia are usually of the order of 30–50% every 24 h having checked for any signs or symptoms of opioid neurotoxicity. Symptoms of neurotoxicity include troublesome confusion or cognitive slowing, misperceptions or visual hallucinations. If neurotoxicity occurs reduce the dose of opioids by 30% and add adjuvant analgesics e.g. gabapentinoid.

Thirty percentage of patients will experience nausea for 3–5 days on starting an opioid. Ensure that an antiemetic is available for use as and when needed e.g. metoclopramide 10 mg per oral (PO) or subcutaneously (SC) three times a day. Patients will experience constipation and need to be prescribed a regular daily laxative. Senna 7.5 mg twice a day is recommended as a starting dose.

Avoid morphine, oxycodone, tramadol or codeine as background analgesia as they can rarely be titrated to effect in this population without opioid-induced neurotoxicity. Once pain is stable, transdermal fentanyl or buprenorphine may be used in place of opioids by continuous subcutaneous infusion. If the lesions heal and pain resolves it is important to reduce the opioids step by step and aim to withdraw them fully.

To address the neuropathic element of pain secondary to calciphylaxis, gabapentin 100 mg after each haemodialysis session or pregabalin 25 mg after each haemodialysis session may be used in conjunction with opioids. If the patient is not on haemodialysis, start with a dose of 100 mg of gabapentin on alternate days or 25 mg of pregabalin on alternate days. Caution is needed as gabapentinoids accumulate in ESRD though they are removed by dialysis [22, 23].

Discuss the use of a non-steroidal anti-inflammatory drug (NSAID) with the nephrology team as they are very effective for the management of the inflammatory aspect of pain secondary to calciphylaxis. If residual renal function is not important, use an oral or parenteral NSAID (e.g. ketorolac) by continuous subcutaneous infusion for pain that is not controlled with the measures outlined above. Cover with a proton pump inhibitor is recommended. In general, NSAIDs are best used at the minimally effective dose and for a short duration but their harms are weighed against their opioid sparing effect [24].

If background pain remains problematic or dose-limiting toxicity is encountered, consider the addition of methadone as a co-analgesic or rotation to methadone from the current opioid. This step may require hospice inpatient care and should only be undertaken with specialist palliative care guidance.

### Breakthrough pain control

Even when background pain control is achieved, the patient may continue to experience spontaneous breakthrough pain. Oxycodone 1-2 mg may be used by oral or by subcutaneous injection on a four hourly to 6 hourly basis. In countries where hydromorphone is available, 0.5 mg of hydromorphone sublingually four hourly is useful.

### Procedure related pain control (during dressing changes and incident volitional breakthrough pain e.g. walking)

Short-acting transmucosal fentanyl preparations have a quick onset and offset of action, which makes them a potentially useful option for dressing changes in patients with calciphylaxis. However, in the UK they are licenced for breakthrough cancer pain and contra-indicated in patients who are opioid naïve. They should be used with caution and with careful patient selection and under the guidance of local pain or palliative medicine teams. An alternative to transmucosal fentanyl, is subcutaneous fentanyl or alfentanil, which have short duration of action. There is a mismatch between the time-action relationship of oral opioids and the time course of breakthrough pain such as that precipitated by dressing changes – the patient may be sleepy for some hours after the procedure.

For dressing changes, use a stepwise approach as follows:

- Step1. Short-acting fentanyl preparation (these are available in nasal, sublingual and buccal formulations and it is important to follow the manufacturer’s guidance).
- Step 2. SC fentanyl or alfentanil (short duration of action).
- Step 3. SC opioid and sedation with subcutaneous midazolam.
- Step 4. Anaesthesia with propofol and fentanyl under anaesthetic supervision in theatre.

The use of a pain tool to record patient reported outcome measures is recommended. The integrated palliative care outcome scale (IPOS) renal is a useful tool though not validated for use in calciphylaxis.

## Supplementary information

**Additional file 1.** Survey Questionnaire.

**Additional file 2.** Salient responses to free text questions.

## Data Availability

The datasets used and/or analysed during the current study are available from the corresponding author on reasonable request.

## Abbreviations

ESRD: End-stage renal disease
NSAID: Non-steroidal anti-inflammatory drug
SC: Subcutaneous
CSCI: Continuous subcutaneous infusion
STS: Sodium thiosulphate

## References

[1] Weenig RH, Sewell LD, Davis MDP, McCarthy JT, Pittelkow MR (2007). Calciphylaxis: natural history, risk factor analysis, and outcome. J Am Acad Dermatol.

[2] Nigwekar SU, Zhao S, Wenger J, Hymes JL, Maddux FW, Thadhani RI (2016). A nationally representative study of calcific uremic Arteriolopathy risk factors. J Am Soc Nephrol.

[3] Polizzotto MN, Bs MB, Bryan T, Bs MB, Ashby MA, Bs MB (2006). Symptomatic Management of Calciphylaxis : a case series and review of the literature. J Pain Symptom Manag.

[4] Vedvyas C, Winterfield LS, Vleugels RA (2012). Calciphylaxis: A systematic review of existing and emerging therapies. J Am Acad Dermatol.

[5] Manickam V, Doherty SL, Malabu UH, Sangla KS, Kan G (2012). Calcific uremic Arteriolopathy on multimodal combination therapy: still unmet goal. Int J Nephrol.

[6] Udomkarnjananun S, Kongnatthasate K, Praditpornsilpa K (2019). Treatment of Calciphylaxis in CKD: a systematic review and meta-analysis. Kidney Int Reports.

[7] Salmhofer H, Franzen M, Hitzl W, Koller J, Kreymann B, Fend F (2013). Multi-modal treatment of calciphylaxis with sodium-thiosulfate, cinacalcet and sevelamer including long-term data. Kidney Blood Press Res.

[8] Galloway PAG, El-Damanawi R, Bardsley V, Pritchard NR, Fry AC, Ojha SK (2015). Vitamin K antagonists predispose to Calciphylaxis in patients with end-stage renal disease. Nephron..

[9] Riemer CA, El-Azhary RA, Wu KL, Strand JJ, Lehman JS (2017). Underreported use of palliative care and patient-reported outcome measures to address reduced quality of life in patients with calciphylaxis: a systematic review. Br J Dermatol.

[10] Rich A, Leach A, Ellershaw J (2001). A case of difficult pain in a patient with chronic renal failure and calciphylaxis. J Pain Symptom Manag.

[11] Green J (2004). Calciphylaxis treated with neurolytic lumbar sympathetic block: case report and review of the literature. Reg Anesth Pain Med.

[12] Brandenburg VM, Sinha S, Torregrosa JV, Garg R, Miller S, Canals AZ (2019). Improvement in wound healing, pain, and quality of life after 12 weeks of SNF472 treatment: a phase 2 open-label study of patients with calciphylaxis. J Nephrol.

[13] Noureddine L, Landis M, Patel N, Moe SM (2011). Efficacy of sodium thiosulfate for the treatment for calciphylaxis. Clin Nephrol.

[14] Olaniran KO, Percy SG, Zhao S, Blais C, Jackson V, Kamdar MM (2019). Palliative Care Use and Patterns of End-of-Life Care in Hospitalized Patients With Calciphylaxis. J Pain Symptom Manag.

[15] Kramann R, Brandenburg VM, Schurgers LJ, Ketteler M, Westphal S, Leisten I (2013). Novel insights into osteogenesis and matrix remodelling associated with calcific uraemic arteriolopathy. Nephrol Dial Transplant.

[16] Murtagh FEM (2018). Palliative Care in Kidney Disease.

[17] Palliative Care Formulary (PCF7). [viewed 2020 Aug 20]. Available from: https://www.pharmpress.com/product/9780857113689/palliative-care-formulary-pcf7.

[18] Davison SN (2019). Clinical pharmacology considerations in pain management in patients with advanced kidney failure. Clin J Am Soc Nephrol.

[19] Caraceni A, Martini C, Zecca E, Fagnoni E. Cancer pain management and palliative care. Handb Clin Neurol. 2012;104:391–415. 10.1016/B978-0-444-52138-5.00027-X 22230457

[20] Sande TA, Laird BJA, Fallon MT (2017). The use of opioids in cancer patients with renal impairment—a systematic review. Support Care Cancer.

[21] King S, Forbes K, Hanks GW, Ferro CJ, Chambers EJ (2011). A systematic review of the use of opioid medication for those with moderate to severe cancer pain and renal impairment: a European palliative care research collaborative opioid guidelines project. Palliat Med.

[22] Randinitis EJ, Posvar EL, Alvey CW, Sedman AJ, Cook JA, Bockbrader HN (2003). Pharmacokinetics of pregabalin in subjects with various degrees of renal function. J Clin Pharmacol.

[23] Bockbrader HN, Wesche D, Miller R, Chapel S, Janiczek N, Burger P (2010). A comparison of the pharmacokinetics and pharmacodynamics of pregabalin and gabapentin. Clin Pharmacokinet.

[24] Wongrakpanich S, Wongrakpanich A, Melhado K, Rangaswami J (2018). A comprehensive review of non-steroidal anti-inflammatory drug use in the elderly. Aging Dis.

