# Supplementary material for: Pain management in patients with end-stage renal disease and calciphylaxis- a survey of clinical practices among physicians
Source: BMC Nephrol. 2020 Sep 18;21:403. doi: 10.1186/s12882-020-02067-2 (PMC7501607; doi:10.1186/s12882-020-02067-2)
Supplement: Supplementary file 1 — Additional file 1. Survey Questionnaire. [file 12882_2020_2067_MOESM1_ESM.docx]

**Additional file 1**

**Survey Questionnaire**

**Survey on knowledge and practices in calciphylaxis pain management**

Calciphylaxis is a rare disease usually seen in dialysis patients. Vascular calcification results in tissue necrosis; pain is a hallmark of this disease and can be extremely difficult to control. Anecdotal data suggests that pain management is variable across the UK but also around the world. This questionnaire aims to gather information on current practice as well as gauging the opinions of specialists involved in the care of patients with calciphylaxis.

The data generated will hopefully enable us to develop recommendations to help clinicians manage calciphylaxis associated pain.

The questionnaire should take no more than 10 minutes.

Q1. In which speciality do you work?

Renal Medicine

Pain Medicine

Palliative Medicine

Q2. Do you work in an acute setting / hospital?

Yes

No

Q3. How many patients with Calciphylaxis / CUA have you seen in the past 10 years?

0

1-2

3-5

5-10

>10

Q4. Do you refer to?( question pertinent to renal team)

Palliative care team

Pain team

Both

Q5 Please describe your current approach to managing pain in Calciphylaxis / CUA?

**Open Text**

Q6 Please list your preferred opioid/s to manage pain in Calciphylaxis / CUA?
 **Choice list**

Number one is your first choice, two is second choice etc.

Background pain – pain that the patient is experiencing over the course of 24 hours. It may vary in intensity.

Breakthrough pain – an exacerbation of more severe troublesome pain that may arise due to movement or may arise spontaneously as a spike in pain over and above the background pain.

Procedure related pain – an exacerbation that occurs with wound management care.

Q7. For background pain do you:

1. Use short acting oral analgesia
2. Use short acting SC analgesia
3. Use modified release oral preparations
4. Use a continuous SC infusion (Syringe Driver)
5. Use a PCA
6. None

Q8. For breakthrough or procedure related pain do you:

1.Use prns oral

2.Use prns SC

3.Use prn Fentanyl e.g. immediate release fentanyl preparations such as lozenge, sublingual or nasal

4.Use PCA

Q9. Please list your preferred adjuvant analgesics to manage pain in Calciphylaxis / CUA?

**Choice list**

Q10. Do you undertake any interventions to manage pain? *(Optional)*

**Open text**

Q11. Does the presence of infection impact on the effectiveness of pain control?

Yes

No

**Reasons – Open Text**

Q12. In your opinion which types of pain are present?

Nociceptive pain

Neuropathic pain

Inflammatory pain

Q13. How often do you see neurocognitive adverse effects when you use opioids to manage pain arising from Calciphylaxis / CUA? *(e.g. misperceptions, hallucinations, myoclonus, seizures)*

Every patient

Most patients

50% of patients

Some patients

Occasional patients

I do not see any adverse effects

Q14. How often do you initiate a conversation about Advance Care Planning with patients with Calciphylaxis?

Never

Rarely

Sometimes

Often

Most patients

All patients

Q15. Please list any tools that you use to measure the effectiveness of pain control? *(Optional)*
