# Supplementary material for: Pain management in patients with end-stage renal disease and calciphylaxis- a survey of clinical practices among physicians
Source: BMC Nephrol. 2020 Sep 18;21:403. doi: 10.1186/s12882-020-02067-2 (PMC7501607; doi:10.1186/s12882-020-02067-2)
Supplement: Supplementary file 2 — Additional file 2. Salient responses to free text questions. [file 12882_2020_2067_MOESM2_ESM.docx]

**Salient responses to free text questions**

**Q5) Please describe your current approach to managing pain in Calciphylaxis / CUA?**

“This is not something that I have come across before and so have not specific management plan, however, I would probably use opioids first line.”

“The patients usually require oxycodone analgesia as the pain is often severe. I refer to the pain team for urgent review and the pain team is also the palliative care team in our hospital.”

“Depends on severity but usually involves escalating opioids - oxycontin short and long acting. Alfentanyl breakthrough and sometimes alfentanyl infusion.”

“Addressing pain around dressing changes - entonox, prn fast acting opiates. Renal analgesic ladder: paracetamol, low doses codeine/tramadol if tolerated, oxycodone IR. On occasional fentanyl patches. Not many patients found benefit from gabapentin anecdotally.”

“Analgesia (WHO ladder), involvement of pain team if required and palliative care if end of life care.”

“Escalating doses of opioids, usually alfentanil. Both cases I have seen have had severe pain and agitated delirium and prognosis measured in days. Anxiolytics and sedative meditation require to palliate agitation.”

“I struggle. Adopt usual approaches with caveat of usually co-existing renal impairment. Have used methadone but without clear benefit. Sometimes NSAID a useful option when no longer any native renal function. Have two patients with severe phantom pain post amputation necessitated by calciphylaxis. Severity and duration of pain prior to surgery presumably contributed to the risk. Can't off hand think of any case where addition of usual neuropathic agents was productive.”

“I would use usual approach to pain management - assess for opiate responsiveness and add trial of adjuvants. However, have very limited experience.”

“Usual titration of paracetamol, Opioids. Early consideration of ketamine - has worked very well in all 3 cases I have been involved in.”

“See what works”

“This is not something that I have come across before and so have not specific management plan, however, I would probably use opioids first line”

“unsure - most recent patient was at the end of life so managed wit opioids / sedatives”

“use analgesic ladder”

“unsure - most recent patient was at the end of life so managed wit opioids / sedatives.”

**Q10. Do you undertake any interventions to manage pain?**

“We would refer to our anaesthetic colleagues”

“Access to interventions with pain team colleagues”

“Would consider femoral nerve block”

“I would refer to the pain management team or palliative care team for their review before considering.”

“Diagnostic epidural”

“Epidural infusion considered in conjunction with the interventional pain services”

“Not personally. We have access to nerve blocks if needed. We have not found these effective, but we do still use them.”

**Q11. Does the presence of infection impact on the effectiveness of pain control? Reasons?**

“don't know, I think so”

“infection reduces effectiveness of analgesia”

“Infection is common in calciphylaxis deposits or ulcers. Always increases pain - management is management if the infection or ischemia”

“often need increase in analgesia background dose”

“Infection/inflammation worsens pain”

“Increases difficulty of managing the pain due to an additional pathogenetic mechanism”

“I would expect this but most I have seen have not been infected”
